# High Incidence of Refeeding Syndrome during the Transition from F75 to Ready-to-Use Therapeutic Feeds among Children 6 to 59 Months with Severe Acute Malnutrition at the Pediatric Nutritional Unit of Mulago Hospital

**DOI:** 10.1155/2024/5469478

**Published:** 2024-09-28

**Authors:** Wani Muzeyi, Teddy Ochieng Andra, Lorraine Oriokot, Victor Musiime

**Affiliations:** Department of Pediatrics and Child Health School of Medicine College of Health Sciences Makerere University, Kampala, Uganda

## Abstract

**Background:**

Refeeding syndrome is a complication developed by children being managed for severe acute malnutrition (SAM). It is caused by changes in electrolyte balance once high-caloric feeding is reinitiated. Phosphorus, potassium, and magnesium are the main electrolytes affected when it occurs. However, hypophosphatemia is the hallmark of the diagnosis of refeeding syndrome. WHO recommends inpatient management of patients with complicated SAM with initially F75 which is low in calories and later transitioned to RUTF which is high in calories but also has a higher phosphorus content.

**Objective:**

This study aims to determine the incidence and factors associated with refeeding syndrome in the transition phase when treating children aged 6 to 59 months with severe acute malnutrition at the Mwanamugimu Nutritional Unit, Mulago.

**Methods:**

We conducted a prospective cohort study at the Mwanamugimu Nutritional Unit of Mulago National Referral Hospital. A total of 150 children between 6 and 59 months with SAM were enrolled into the study. We measured serum electrolytes (phosphorus, sodium, and potassium) at admission, initiation of RUTF, and 48 hours after transition. The refeeding syndrome was diagnosed by a drop in serum phosphorus of more than 0.3 mmol from baseline. The data were analyzed using STATA 17.0. Incidence of refeeding syndrome was determined as the proportion of participants whose serum phosphorus declined by more than 0.3 mmol from baseline. For factors associated, a multivariate-modified Poisson regression analysis reporting relative risk was performed with a 0.2 level of significance at bivariate and 0.05 at multivariate analyses.

**Results:**

Of the 150 children recruited, 35 were lost to follow-up and 115 children had their data analyzed. Of the 115 participants in the study, 40 developed refeeding syndrome indicating a cumulative incidence of 34.8% with a 95% CI of 26.5–44%. A low baseline serum sodium (RR: 0.89, 95% CI: 0.80–0.99, and *P* value: 0.038) and having edematous malnutrition (RR: 0.90, 95% CI: 0.81–0.99, and *P* value; 0.042) at admission were found to be significant (*P* < 0.05) risk factors of refeeding syndrome.

**Conclusion:**

The cumulative incidence of RFS of 34.8% is very high. RFS is found to be associated with low baseline sodium and pedal edema. Children with a low baseline sodium and edema should undergo a cautious transition of feeds.

## 1. Background

Refeeding syndrome (RFS) is a complication developed by children being managed for severe acute malnutrition (SAM) [1].

It is caused by changes in the electrolyte balance once high-caloric intake is reinitiated. In RFS, the body is in an anabolic state and insulin release increases cellular uptake of electrolytes such as potassium, magnesium, and phosphorus [2]. This leads to hypophosphatemia, hypokalemia, and hypomagnesemia and is associated with high morbidity and mortality. Hypophosphatemia has been defined as the hallmark of refeeding syndrome in many studies that have been carried out [2, 3]. The presentation of these biochemical changes is not specific and mimic sepsis [4].

The global incidence of RFS is not known due to the lack of a universal definition as different studies used different cutoffs or drops in phosphorus levels. Several studies have been carried out to look for the incidence of RFS following the initiation of feeds with F75 with subsequent transition to F100. The incidence of RFS has varied from 15% in South Africa, 17% in India, and 21% among Kenyan children with SAM [5, 6].

In 2013, the World Health Organization recommended that children with SAM be transitioned directly to ready to use therapeutic feeds' RUTF order to allow for rehabilitation to continue at home [1].

This study was designed to determine the incidence of refeeding syndrome and its risk factors among children admitted to the nutritional unit of Mulago National Referral Hospital who initiated on F75 feeds and subsequently transitioned to RUFT.

## 2. Materials and Methods

### 2.1. Study Design and Setting

This prospective cohort study was conducted at the Mwanamugimu Nutrition Unit (MNU) in Mulago National Referral Hospital, Kampala, from September to December 2022.

All children with SAM are admitted to the MNU through the acute care unit (ACU) which is the pediatric medical emergency department where initial nutritional assessment is conducted and children with SAM are given emergency care and also started on F75 130 ml/kg/24 hrs that is given in 2 hours doses, urgent investigations are done, and the children are sent to MNU.

The nutritional unit of Mulago Hospital is an 80-bed capacity unit, and on average it receives about 100 children with severe acute malnutrition per month. The unit has five divisions, the high dependence unit (HDU) in which very sick children with SAM from the ACU are admitted for the continuation of emergency care and the stabilization phase for noncritical. There is also the transition phase locally known as P.2, the rehabilitation phase locally known as P.3, and the outpatient departments.

At the MNU, all children received from the ACU are reclerked by an intern medical officer who takes a thorough history and examination including the nutritional assessment, taking note of all medical complications and then decides whether the child is admitted to HDU or is admitted to the stabilization unit. The F75 feeds are continued and management of medical complications.

Upon stabilization of medical complications, reduction of edema, and return of appetite (all children undergo an acceptance), the children are transitioned to RUTF and then moved to the transition phase where they are monitored for refeeding syndrome. Children who deteriorate while in the transition or rehabilitation phase are usually returned to the stabilization phase and F75 restarted.

Children who are stable after 48 hours in the transition phase on RUTF are then graduated to the rehabilitation phase where they are given ekitoobero (high energy and nutrient-dense feeds), given nutritional education, and then discharged home and reviewed weekly at the outpatient therapeutic clinic until resolution of malnutrition.

### 2.2. Sample Size

A sample size of 132 participants was calculated based on the Kelsey formula for two proportions assuming a 95% level of confidence, 80% power, and also taking into account that 25% of children with shock (exposure) developed refeeding syndrome compared to just 7% among those that did not have refeeding syndrome [5].

### 2.3. Study Procedure

Participants with SAM were enrolled in the study at the ACU, where data on demographic and clinical variables were collected using a structured questionnaire and baseline investigations such as electrolytes (phosphorus, potassium, and sodium) and complete blood count, blood smear for malaria parasites among others were determined before initiation of feeds. The electrolytes were repeated at 48 hours into the transition (RUTF feeds). The blood sample for electrolytes was collected using venous blood (2.5 ml) into a plain vacutainer (red top).

To minimize variability in the sample collection and analysis, a standardized protocol was used for sample collection, storage, and analysis. A senior phlebotomist was used to collect the samples and transfer them to the main laboratory of Mulago National Referral Hospital where the samples were analyzed by a senior laboratory technologist. The samples were run within 30 minutes of collection using the COBAS 6000 chemistry machine, and results were checked by the PI who identified abnormal results and these were rerun for confirmation.

### 2.4. Statistical Analysis

A structured questionnaire was used to collect data. Completed questionnaires were entered into a computer using Epidata and exported to STATA 17.0 for analysis. RFS was defined as a phosphorus drop of 0.3 mmol/L or more from the baseline value. To assess for independent associations of refeeding syndrome, we used the modified Poisson regression with robust standard errors. Variables with *P* value < 0.2 were considered for the multivariate analysis where factors with a *P* value less than 0.05 were considered to be risk factors for refeeding syndrome. Significant variables were tested for interaction, and dropped variables were tested for confounding.

## 3. Results

### 3.1. Study Profile

**Figure 1:**
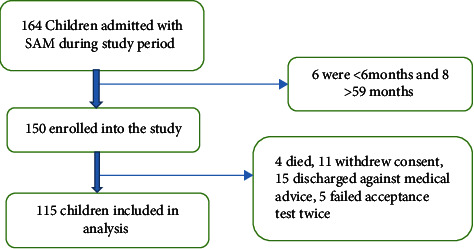
Study flowchart.

Of the 150 children that were enrolled in the study, 35 were lost to follow-up. This is summarized in Figure 1 below.

### 3.2. Participant's Characteristics

The mean Muac in the study was 10.64, the majority (55.7%) of the participants were over 12 months, and over 53% of the participants had edematous malnutrition. The results are summarized in Table 1 below.

### 3.3. Incidence of Refeeding Syndrome

Of the 115 participants in the study, 40 had a phosphorus drop of 0.3 mmol/L or more from the baseline value and were therefore taken to have developed refeeding syndrome, indicating a cumulative incidence of 34.8% with 95% CI of 26.5–44.1.

### 3.4. Risk Factors of Refeeding Syndrome

Malaria positivity and edematous malnutrition were found to be associated (*P* < 0.2) with refeeding syndrome at the bivariate analysis. The results are summarized in Table 2 below.

### 3.5. Baseline Serum Laboratory Findings among Study Participants

A low baseline serum sodium was associated with refeeding syndrome at bivariate. The results are summarized in Table 3 below.

### 3.6. Factors Associated with Refeeding Syndrome

Low baseline serum sodium and edematous malnutrition were found to be significant (*P* value < 0.05) risk factors for developing refeeding syndrome. This is summarized in Table 4 below.

## 4. Discussion

This study aimed to determine the incidence and factors associated with refeeding syndrome in the transition phase of treating children with SAM aged 6 to 59 months admitted to the MNU, Mulago.

### 4.1. Incidence of Refeeding Syndrome

The incidence of RFS among children aged 6 to 59 months at the MNU, Mulago Hospital, was 34.8% (95% CI26.5-44.1). A prospective cohort study in India measuring phosphorus at admission, end of stabilization, and at discharge by Dakshayani et al. in 2019 found the incidence of RFS to be 31% in transition [3]. This value falls within the 95th CI of our study suggesting that the incidence of RFS is quite high globally with little variation across continents.

When compared to a study carried out in South Africa by Mbethe and Mda in 2017 found the incidence of RFS to be 15% [5]. Their lower incidence can allude to the exclusion of very ill children in their study, and also, they only followed the children for 5 days from admission.

### 4.2. Factors Associated with Refeeding Syndrome

The refeeding syndrome was found to be associated with low baseline sodium. Alterations in carbohydrate metabolism have a profound effect on sodium water balance [7]. The hallmark of RFS is derangements of electrolytes particularly sodium, potassium, phosphate, and magnesium [4].

Low baseline sodium predisposes to refeeding syndrome because an increase in sodium pump activity due to a rapid increase in supply of energy causes rapid increase of accumulated sodium from cells causing extracellular expansion and this contributes to the worsening edema that develops during refeeding syndrome [8]. In a study on the incidence of refeeding syndrome and its associated factors in South Africa, RFS was found to be associated with baseline hypocalcemia, hypokalemia, and hypomagnesemia [5].

However, these associations were only at the bivariate analysis using Fisher's exact test, perhaps further analysis at multivariate would have excluded them. In our study, we did not measure baseline calcium and magnesium.

Edematous SAM at admission was found to be significantly (*P*=0.042) associated with refeeding syndrome in our study. Children with SAM edema have been reported to have poor outcomes compared to those with nonedematous malnutrition [9, 10]. It is therefore unsurprising that pedal edema was associated with RFS. This is similar to the findings in another study in South Africa on refeeding syndrome, where RFS was found to be associated with edema at admission. Carbohydrate introduction to a diet leads to a rapid decrease in renal excretion of sodium and water. Institution of fluid to maintain normal urine output leads to fluid overload, thus edema, congestive cardiac failure, cardiac arrhythmias, and pulmonary edema [11].

This study should be interpreted in light of some limitations. The desire sample size was not achieved due to participants' loss to follow-up, and this may have introduced bias and affect the generalizability of the study. The follow-up period was limited to 48 hours, and as a result, the study was not able to capture long-term outcomes of refeeding syndrome.

## 5. Conclusion

The incidence of refeeding syndrome was high where 34.8% of the children admitted with SAM developed refeeding syndrome during the transition from F75 to RUTF. Low baseline sodium and edematous SAM were significant risk factors for refeeding syndrome.

## 6. Recommendations

Children with SAM should be assessed for baseline sodium levels. Those found to have low baseline serum sodium and or having edematous SAM should be cautiously transitioned from F75 to RUTF to prevent development of refeeding syndrome.

## Figures and Tables

**Table 1:** Characteristics of 115 study participants.

Variable	Frequency	Percentage
Age (months)		
≤12	51	44.3
>12	64	55.7
Muac		
Mean ± SD	10.64 ± 1.53	
Fever		
No	105	91.3
Yes	10	8.7
Oral thrush		
Yes	26	22.6
No	89	77.4
Vomiting		
Yes	37	32.2
No	78	67.8
Diarrhea		
Yes	58	50.5
No	57	49.6
Pedal edema		
Yes	62	53.9
No	53	46.1
Dermatoses		
Yes	53	46.1
No	62	53.9
Malaria		
Positive	4	3.5
Negative	111	96.5

**Table 2:** Bivariate analysis of factors associated with refeeding syndrome.

Variable	Reseeding		Crude RR	95% CI	*P* value
	Yes	No			
Age (months)					
≤12	18 (45.0)	33 (44.0)	1.00		
>12	22 (55.0)	42 (56.0)	1.00	0.90–1.12	0.919
Muac					
Mean ± SD	10.8 ± 2.0	10.6 ± 1.2	0.99	0.95–1.02	0.466
Fever					
No	37 (92.5)	68 (90.7)	1.00		
Yes	3 (7.5)	7 (9.3)	1.03	0.86–1.23	0.729
Oral thrush					
Yes	6 (15.0)	20 (26.7)	1.00		
No	34 (85.0)	55 (73.3)	0.91	0.82–1.02	0.115
Vomiting					
Yes	14 (35.0)	23 (30.7)	1.00		
No	26 (65.0)	52 (69.3)	1.03	0.92–1.15	0.642
Diarrhea					
Yes	20 (50.0)	38 (50.7)	1.00		
No	20 (50.0)	37 (49.3)	0.99	0.89–1.11	0.946
Edema					
Yes	16 (40.0)	46 (61.3)	**1.00**		
No	24 (60.0)	29 (38.7)	**0.89**	**0.80–0.99**	**0.03**
Dermatoses					
Yes	18 (45.0)	35 (46.7)	1.00		
No	22 (55.0)	40 (53.3)	0.99	0.89–1.10	0.865
Malaria					
Positive	3 (7.5)	11 (14.7)	**1.00**		
Negative	37 (92.5)	64 (85.3)	**0.91**	**0.80–1.05**	**0.193**

All variables with *P* value <0.2 at bivariate are significant at bivariate and were put in bold.

**Table 3:** Baseline laboratory findings of study participants.

Variable	Refeeding		Crude PR	95% CI	*P* value
	Yes	No			
Potassium (mmol/L)	**5.4** **±** **0.9**	**5.4** **±** **3.2**			
Low (<3.5)			1.00		
Normal			0.9	0.65–1.26	0.549
Sodium (mmol/L)	**139.2** **±** **3.6**	**137** **±** **5.3**			
Low (<135)			1.00		
Normal			0.86	0.78–0.96	**0.005**
Hemoglobin (g/dL)	**9.3** **±** **1.7**	**9.6** **±** **3.03**			
Low (<11.0)			1.00		
Normal			1.00	0.99–1.02	0.402

All variables with *P* value <0.2 at bivariate are significant at bivariate and were put in bold.

**Table 4:** Factors associated with refeeding syndrome.

Variable	Crude RR	Adjusted RR	95% CI	*P* value
Baseline Sodium (mmol/L)				
Low	1.00	1.00		
Normal	0.86	0.88	0.79–0.98	0.018
Edematous SAM				
Yes	1.00	1.00		
No	0.89	0.9	0.81–0.99	0.041
Malaria test				
Positive	1.00	1.00		
Negative	0.91	0.91	0.80–1.05	0.196

## Data Availability

All data used to support the findings of this study are attached as a supplementary data file.

## Supplementary Materials

Supplementary Materials An excel sheet of the study data set has been attached as a supplement.
