# Supplementary material for: High Incidence of Refeeding Syndrome during the Transition from F75 to Ready-to-Use Therapeutic Feeds among Children 6 to 59 Months with Severe Acute Malnutrition at the Pediatric Nutritional Unit of Mulago Hospital
Source: J Nutr Metab. 2024 Sep 28;2024:5469478. doi: 10.1155/2024/5469478 (PMC11455593; doi:10.1155/2024/5469478)
Supplement: Supplementary Materials — An excel sheet of the study data set has been attached as a supplement. [file 5469478.f1.pdf]

Study_Nun	Age	Sex	Muac	Birth_order	Chroinc_co	Specifiy	refferal	Immunisati
8	23	1	12	3	2			1
9	7	1	10.5	1	2		2	4
10	17	1		2	2		2	1
11	13	1	11.2	1	2		1	1
12	9	1	11	1	2			1
13	12	2	8.8	1	1	cerebral palsy		2
17	16	1	10.9	6	2			4
18	16	1	10.5	5	2			4
19	11	2	7.4	1	1	CLEFT LIP		2
22	6	1	7.3	3	2			1
23	18	1	11.5	3	2			1
24	14	1	10.5	1	2			1
25	12	1	11	2	2			1
26	18	2	12	2	2			2
27	10	1		1	2			2
29	12	1	11	4	2			2
30	13	1	9.8	4	2			2
31	13	1	9.5	3	2			2
32	15	2	10.7	3	2			2
36	18	1	11.3	4	2			3
39	13	1	10	3	2			3
40	14	2	10.4	2	2			3
41	18	1	10	2	2			3
42	13	1	11.4	5	2			3
43	9	1	12	4	2			3
45	10	2	12	1	2			3
46	12	2	9.2	3	2			3
47	36	2	11.6	1	2			3
48	9	1	11.6	2	2			2
49	9	1	11.2	2	2			2
1	19	2	9.4	5	2			2
2	18	2	11	2	2			1
3	8	1	9.5	2	2			2
4	14	1	9.6	1	2			2
5	23	1	13.1	1	2			3
6	23	2	11.2	2	2			2
7	21	1		1	2			4
14	9	1	8.7	2	2			2
15	32	2	10	2	2			2
16	6	1	12	5	2			2
20	11	1	11.9	1	2			1
21	18	2	9.4	3	2			2
28	21	2	10.1	3	2			3
33	13	2	10.9	1	2			2
34	7	2	8.2	7	1	CHD-largeVSD		1
37	2	2	10.4	5	1	hydrocephalus		2

35	27	2	11.2	6	1	CP, Hydrocephalus		2
38	6	2	10.5	4	2			2
42	12	2	11	2	2			3
44	9	1	11	5	2			3
51	18	1	11.3	4	2			3
52	36	1	11	1	2			3
53	13	2	10.9	2	2			2
54	16	2	11	1	2			3
55	12	2	8.1	3	2			2
56	18	2	11.2	2	2			3
57	9	2	10	2	2			2
58	46	1	9.2	2	2			3
59	21	1	8.9	1	2			3
60	42	2	12.3	2	2		2	2
61	8	1	10.6	2	2			2
62	24	2	10.2	1	2			2
63	8	2	12	1	2			2
64	11	1		2	2			2
65	9	2	11.8	1	2			2
66	12	2	11.8	1	2			3
67	6	1	21	5	2			2
68	14	2	12.8	1	2			3
69	8	1	10.8	4	2			2
70	9	2	11.1	1	2			3
71	23	2	11.2	6	2			2
72	14	1	9.3	1	2			3
73	8	2	10.4	2	2			1
74	17	1	8.8	4	2			3
75	48	1	11.4	5	1	paed hiv		3
76	34	1	12.4	1	1	cerebral palsy		3
77	15	2	9.2	1	2			1
78	20	1	14.5	4	2			3
79	7	1	9.5	2	2			2
80	12	1	12	3	2			2
81	36	1	13.5	3	2			1
82	9	1	9.8	4	2			3
83	9	2	10	1	2			2
84	13	2	11.3	2	2			2
85	11	1	10.2	1	2			3
86	10	1	10.2	8	2			2
87	12	1	12	2	2			2
88	12	2	12.5	1	2			2
89	14	2	10.3	2	2			1
90	14	2	10	3	2			4
91	7	2	10.8	1	1	cerebral palsy		2
92	25	1	10.2	3	2			3
93	12	1	9.8	3	2			3

94	44	1	12.2	2	2		3
95	24	2	11.7	1	2		2
96	15	2	10.2	8	2		1
97	22	2	12	2	2		3
98	13	2	11.2	5	2		2
99	48	2	10.2	2	2		1
100	33	1	11.2	7	2		3
101	14	2	9	2	2		3
102	12	2	9.6	3	2		3
103	9	1	10	1	2		3
104	48	1	11.4	1	2		3
106	18	1	10.2	5	2		2
107	9	2	9.2	2	2		3
108	30	1	8.7	9	2		2
109	14	2	9.6	3	2		3
110	10	2	10.2	3	2		2
111	13	1	10.2	1	2		3
112	11	1	10.1	1	2		2
113	11	1	11.2	2	2		2
114	7	1	9.4	5	2		2
116	7	2	9.2	2	2		2
117	12	1	10.3	2	2		3
118	12	1	10.8	3	2		3
119	24	2	8.6	3	2		3
120	24	2	11.2	3	2		3
121	36	2	9.6	2	2	cerebral palsy	3
122	9	1	9.8	3	2		2
123	6	2	10.2	2	2		2
124	36	1	9.6	6	2		3
125	12	1	10.2	3	2		2
126	13	1	9.8	2	2		2
127	24	2	9.7	2	2		4
128	8	1	8.5	1	2		2
129	18	2	9.8	2	2		3
130	17	2	11.2	2	2		3
132	18	1	11.2	5	2		3
141	10	1	9.8	4	1	downs syndrome, CHD	2
144	16	1	9.8	1	2		3
136	15	1	10.8	2	2		3
105	24	1	8.9	3	2		2
133	16	2	11.2	1	2		2
115	11	2	8.9	2	2		2
138	8	2	9	2	2		2
139	18	1	11.2	3	2		3
131	36	2	11.2	7	2		3
143	24	2	11.8	4	2		3
140	12	1	10.8	5	2		3

148	21	1	9.2	2	2	1
147	11	1	9.8	1	2	2
149	12	1	10.2	3	2	2
150	16	1	9.8	3	2	1
134	30	2	11.2	0	2	4
135	17	2	9.6	1	2	1
142	18	2	9.6	2	2	3
145	12	1	10.6	3	2	2
137	7	1	9.8	2	2	2
146	7	2	9.8	2	2	2

Temperatu	Oral_thrusl	LOC	Vomiting	Diarrhoea	Dehydratio	Congenital	Specify	Edema
36	2	1	2	2	2	2		1
36.5	2	1	2	1	2	2		2
36.2	2	1	2	2	2	2		1
36	2	1	2	2	2	2		1
36	2	1	2	1	2	2		2
37.3	2	1	1	2	2	2		2
36.3	2	1	2	1	2	1	polydactyl	2
36.5	2	1	2	1	2	2		1
36.2	2	1	2	2	2	1	CLEFT LIP	2
36.5	2	3	2	2	1	2		2
38.4	2	1	2	2	2	2		1
37.2	2	1	1	1	2	2		1
36.7	2	3	1	2	1	2		2
36.8	2	1	1	2	2	2		1
36.8	2	1	2	2	2	2		1
36.6	1	1	1	1	2	2		2
37.6	1	1	2	1	2	2		2
5	1	1	2	2	2	2		2
36.2	1	1	2	1	2	2		1
35.7	2	1	2	2	2	2		1
36.9	2	1	2	1	2	2		2
37.2	1	1	2	2	2	2		2
37.8	2	1	2	1	2	2		2
36.8	2	1	2	1	2	2		1
37.2	2	1	2	1	2	2		1
36.2	2	1	2	2	2	2		1
37.2	2	1	2	1	2	2		1
38	2	1	1	1	2	2		1
36.7	1	1	1	1	2	2		2
36.8	2	1	1	1	2	2		2
36	2	1	2	2	2	2		1
36.5	1	1	2	2	2	2		1
36.4	2	1	2	2	2	2		2
36.2	2	1	1	1	2	2		1
36.1	2	1	2	2	2	2		1
38.1	2	1	2	2	2	2		2
36.4	2	1	2	2	2	2		2
35.6	2	1	2	2	2	2		2
36.2	1	1	1	1	2	1	imperforat	2
36.2	2	1	1	2	2	2		1
36	1	1	2	2	2	2		1
36.5	2	1	2	1	2	2		2
36.8	2	1	2	2	2	2		2
36.8	1	1	1	1	1	2		2
36.4	2	1	2	1	2	2		2
37.2	2	1	2	2	2	1	hydroceph	1

36.5	2	1	2	2	2	1	hydrocepha	1
37.6	2	1	1	1	2	2		2
37.2	2	1	2	1	2	2		2
36.2	2	2	2	1	2	2		2
37.8	1	1	1	2	2	2		2
36.2	2	1	2	2	2	2		1
36.8	2	1	2	1	2	2		2
37.8	2	1	1	1	2	2		2
36.2	1	1	2	1	2	2		1
36.2	1	1	1	1	2	2		2
36.2	2	1	2	1	2	2		2
37.2	2	1	2	2	2	2		1
36.8	1	2	1	1	1	2		2
36.8	2	1	2	2	2	2		1
36.2	2	1	2	2	2	2		2
36.8	2	1	2	2	2	2		2
37.2	2	1	2	1	2	2		2
36.8	2	1	2	2	2	2		2
37.2	2	1	1	1	2	2		2
36.7	2	1	2	2	2	2		2
37.8	2	1	1	1	2	2		2
36.7	2	1	1	1	2	2		1
36.2	2	1	2	2	2	2		2
36.7	2	1	2	2	2	2		2
36.1	2	1	2	2	2	2		1
36.8	2	1	2	2	2	2		2
36.3	2	1	2	2	2	2		2
36.5	1	1	2	2	2	2		2
36.7	1	1	2	2	2	2		1
36.2	1	4	2	2	2	1	dump and [illegible]	2
36.8	2	1	2	2	2	2		1
39.1	2	1	2	1	2	2		1
36.3	2	1	2	1	2	2		2
36.2	1	1	2	1	2	2		1
36.4	2	1	2	2	2	2		1
36.8	2	1	1	1	2	2		2
37.8	2	1	1	1	2	2		1
36.1	2	1	2	2	2	2		2
36.2	2	1	1	1	2	2		1
36.2	2	1	2	2	2	2		2
36.8	2	1	2	1	2	2		1
36.2	2	1	2	2	2	2		2
36.2	2	1	2	2	2	2		2
36.6	2	1	1	1	2	2		2
36.6	2	1	2	1	2	1	cerebral pa	2
36.7	1	1	1	2	2	2		1
36.8	2	1	1	1	2	2		1

36.2	1	1	2	1	2	2		1
35.9	2	1	2	2	2	2		1
35.1	2	1	2	2	2	2		2
36.2	1	1	2	1	2	2		2
36.9	2	1	2	2	2	2		2
36.3	2	1	2	2	2	2		2
36.7	2	1	1	2	2	2		1
36.7	2	1	1	1	2	2		1
36.6	1	1	1	1	2	2		2
36.1	2	1	1	2	2	1	cerebral pa	2
36.2	1	2	2	1	2	1	cerebral pa	2
36.7	2	1	2	2	2	2		1
36.2	2	1	2	2	2	2		1
36.8	1	1	2	1	2	2		1
36.7	1	1	1	2	2	2		1
36.7	2	1	1	1	2	2		2
36.6	2	1	1	2	2	2		1
36.8	2	1	1	1	2	2		1
36.2	1	1	1	1	2	2		1
36	2	1	1	2	2	2		1
37	2	1	1	2	2	2		1
36.4	2	1	1	1	2	2		1
36.4	2	1	2	1	2	2		2
36.2	2	1	1	1	2	2		1
37	1	1	2	2	2	2		1
36.2	2	1	2	2	2	1	cerebral pa	2
37	2	1	1	1	2	2		2
36.2	1	1	2	2	2	2		1
36.7	2	1	2	2	2	2		1
37	2	1	2	1	2	2		1
36.6	1	1	2	1	2	2		1
36.6	2	1	2	2	2	2		1
36.1	2	1	2	2	2	2		1
36.2	1	1	2	2	2	2		2
37	2	1	2	1	2	2		1
36.2	2	1	2	2	2	2		1
36.9	2	1	2	1	2	1	downs sync	1
36.2	2	1	1	1	1	2		1
37.7	2	1	2	1	2	2		1
37.2	2	2	2	1	2	1		1
36.1	2	1	2	2	2	2		2
36.5	1	1	1	1	2	2		2
36.4	2	1	2	1	1	2		1
36.5	2	1	2	1	2	2		1
36.7	2	1	1	2	2	2		1
36.7	2	1	2	1	2	2		1
36.8	1	1	2	2	2	2		1

36.2	1	1	2	1	2	2		1
36.3	2	1	1	2	2	2		1
37.8	2	1	1	1	2	2		1
36.8	2	1	2	1	2	2		1
36.7	2	1	2	1	2	2		1
37	2	1	2	1	2	2		1
36.8	1	1	1	1	2	2		1
36.7	2	1	2	1	1	2		1
36.8	2	1	1	1	2	2		1
36.1	1	1	2	1	2	2		1

Wastig	CRT	Hepatomeg	dermatose	breastfeed	breastfeed	NGT	Phosphoru	Phosphoru
2	2	2	2	12	2	2	4.7	4.5
1	2	2	2	0	2	2	4.45	4.8
2	1	2	2	6	2	2	4	4.4
2	1	2	2	7	2	2	6.5	5.3
2	1	2	2	9	1	2	4.9	5.7
1	1	2	2	6	2	1	4	4.3
1	1	2	1	1	2	1	6.4	6
2	1	2	1	2	2	2	4.6	5
1	1	2	2	2	2	1	4.7	4.6
1	2	2	2	6	1	2	2.9	2.8
2	2	2	2	12	2	2	4	4.3
2	3	2	2	12	2	1	4.5	3.7
1	3	2	2	3	2	1	4.4	4.6
1	1	2	1	14	2	2	3.3	4.5
2	3	2	1	0	2	2	2.1	2.8
1	1	2	1	4	2	1	2.2	3.9
1	2	2	2	12	2	2	4.6	4.8
1	2	2	2	12	2	2	5.8	4
1	1	2	1	6	2	2	3.2	4.4
2	2	2	1	11	2	2	5.4	3.3
1	2	2	2	13	1	2	2.1	6.3
1	1	2	1	14	1	2	3.8	5.2
1	2	2	2	12	2	2	3.4	6.6
1	2	2	1	8	2	2	4.3	4.4
1	1	2	2	9	1	2	5.8	5.1
1	1	2	1	8	2	2	3.2	3.9
1	2	2	1	12	2	2	4.8	3.7
1	1	2	1	18	2	2	5.6	4.6
1	2	2	2	9	1	2	4.6	6.8
1	2	2	2	9	1	2	4.8	4.4
1	2	2	2	12	2	2	3.2	
1	2	2	1	9	2	2	4.4	
1	1	2	2	2	2	2	5.8	
1	2	2	2	10	2	2		
1	1	2	2	6	2	2	4.5	
1	3	2	2	15	2	2	2.3	
2	1	2	2	13	2	2	5.7	
1	2	2	2	9	1	1	4.6	
1	1	2	2	8	1	2	2.3	
1	1	2	1	4	2	1	4.8	
2	3	2	2	4	2	2	6.1	
1	3	2	2	7	2	2	4.4	
1	1	2	2	12	2	2	6.3	
1	2	2	1	12	2	2	2.4	
1	2	2	2	7	1	2	5.5	
2	2	2	1	17	1	1	4.7	

2	2	2	1	6	2	1	3.4	
1	2	2	2	6	1	2	5.5	
2	1	2	2	12	1	2	4.3	4.4
1	1	2	2	9	1	2	4.8	
1	2	2	2	14	2	2	2.3	2.7
1	1	2	1	7	2	2	5.4	6
1	2	2	2	6	2	2	6.1	3.3
1	2	2	2	4	2	2	2.5	2.9
1	2	2	1	3	2	2	3.3	1.8
1	2	2	1	12	2	2	5.6	4.7
1	2	2	2	8	2	2	4.6	
1	2	2	2	24	2	2	3.5	4.6
1	2	2	1	18	2	2	3.1	
2	2	2	1	2	2	2	3	3.3
1	2	2	2	6	2	2	7.6	3.1
1	2	2	2	12	2	1	5.8	5.3
1	2	2	2	8	1	2	3.8	6.2
2	2	2	2	11	1	2	3.1	5.1
2	2	2	1	9	1	2	6	5.5
1	2	2	2	12	1	2	3.9	3.7
1	2	2	2	6	1	2	5.7	4.7
1	2	2	1	8	2	2	3.3	3.9
1	2	2	1	4	2	2	6.3	5.2
1	2	2	2	8	2	2	3.9	4.5
2	1	2	1	5	2	2	4.3	3.3
1	2	2	2	14	1	2	5.2	5.8
1	1	2	1	4	2	2	4.4	4.9
1	2	2	1	16	2	2	3.4	5.3
1	2	2	1	18	2	2	6.2	4.1
1	2	2	2	5	2	1	5.7	6.1
1	1	2	1	12	2	2	4.8	5.5
1	2	2	2	18	2	2	2.1	3
1	2	2	2	2	2	2	4.8	4.6
1	2	2	2	8	2	1	4.1	4.9
2	2	2	1	12	2	2	4.7	1.4
1	2	2	1	9	1	2	4.8	4.6
1	2	2	2	0	2	2	3.7	3.9
1	2	2	1	13	1	2	7.3	5.9
1	2	2	1	1	2	2	4.7	4.6
1	1	2	2	2	2	2	6.6	5.3
1	1	2	1	7	2	2	5.3	5.5
2	2	2	2	3	2	2	8.1	6
1	2	2	2	3	2	2	6.6	6.1
1	2	2	2	3	2	2	6.5	6.3
2	1	2	1	7	1	2	5.7	5.8
1	2	2	1	8	2	2	6.1	5.9
1	1	2	1	6	2	2	4.6	4.7

1	1	2	1	12	2	2	4.6	4.1
2	2	2	1	12	2	2	4.8	5
1	1	2	1	3	2	2	5.6	5.9
1	1	2	1	20	2	2	3.3	4.7
1	2	2	1	4	2	2	4.3	3.5
1	1	2	1	4	2	2	6.8	6.6
2	1	2	1	24	2	2	5.5	4.8
1	2	2	2	10	2	2	5.6	5
1	2	2	2	10	2	2	5.5	4.8
1	2	2	2	9	2	2	3.6	5.1
1	2	2	1	18	2	2	4.4	3.9
1	2	2	2	6	2	2	4.9	5.4
1	2	2	1	4	2	2	6.8	4.6
1	2	2	1	0	2	2	1.8	3.3
1	1	2	2	9	2	2	5.3	5.9
1	2	2	2	5	2	2	3.7	4.5
1	1	2	2	12	2	2	5.2	5
2	2	2	1	9	2	2	4	4
1	1	2	1	6	2	2	4.7	4.7
1	1	2	2	1	2	2	5.5	5.9
1	2	1	1	4	2	2	6	
1	1	2	2	11	2	2	4.7	4.9
1	1	2	2	12	1	2	6.2	5.2
2	2	2	1	8	2	2	3.4	4.7
2	1	2	1	12	2	2	2.8	4.5
1	2	2	2	14	2	2	3.6	4.2
2	2	2	2	6	2	2	4.3	5.9
1	1	2	2	6	2	2	5.1	6
1	2	2	1	18	2	2	5	5.6
1	1	2	1	6	2	2	4.8	4.9
1	2	2	1	6	2	2	4.6	
1	2	2	1	14	2	2	4.2	5.9
2	1	2	2	6	2	2	4.2	5.8
1	1	2	1	16	2	2	4.4	5.4
1	2	2	2	3	2	2	6.8	6.9
1	2	2	1	12	2	2	5.2	5.9
2	2	2	2	10	1	2	5.4	
1	2	2	2	8	2	2	4	
2	1	2	2	12	2	2	2.7	
1	2	2	1	9	2	2	1.7	
2	1	2	2	16	2	2	4.8	
2	1	2	2	6	2	2	6	
1	1	2	1	8	1	2	4.2	
2	1	2	1	14	2	2	5.6	
1	2	2	1	4	2	2	4.3	
1	1	2	2	6	2	2	3.9	
1	2	2	1	4	2	1	3.6	

1	2	2	2	12	2	2	2.4	2.6
1	3	2	2	6	2	2	4.1	4.1
1	1	2	2	8	2	2	5	4.8
1	1	2	2	12	2	2	5.5	5.5
2	1	2	2	18	2	2	4.2	4.2
1	1	2	1	14	2	2	4.1	4.6
1	2	2	1	12	2	2	3.8	4.8
1	2	2	2	6	2	2	3.9	3.9
1	2	2	2	4	2	2	6	4.6
2	2	2	2	5	2	2	5.2	

Phosphoru	pottasium1	pottasium2	pottasium3	Sodium1	sodium2	Sodium3	Haemoglob	Malaria
4.4	6.2	5.45	5.53	140	138	141	10.2	2
4.6	6.8	6.3	6.25	141	141	141	9.8	1
5.2	6.24	5.31	5.22	141	137	141	8.9	1
5.4	6.1	5.45	5.35	145	141	144	10	1
4.5	5.02	7.43	7.32	139	136	138	9.8	1
4.1	3.14	6.29	5.26	125	137	136	8.4	1
5.4	5.38	5.38	4.74	139	138	142	9.8	2
4.6	5.22	5.78	5.77	136	139	139	10.8	2
4.8	5.74	6.4	5.28	138	143	141	9.2	2
7.2	4.37	5.5	8.27	135	139	134	8.6	1
4	3.6	4.95	4.35	137	143	141		1
4.3	5.55	5.12	5.28	141	140	142		1
4.9	4.1	5.25	5.75	138	138	139	9	1
5	4.59	4.82	5.25	138	142	140	4.4	1
3.2	3.92	4.65	5.6	141	142	147	7.8	2
4.1	6.25	4.12	4.27	129	138	141	9.2	2
5.4	3.9	5.27	4.98	135	138	138	9.2	2
4.9	4.37	5.4	5.12	142	138	139	10	2
4.6	4.15	5.01	5.14	140	142	141	9.8	2
4.2	4.89	5.53	7.35	144	139	139	10.8	2
6.4	5.19	4.32	4.2	134	137	137	3.5	1
5.1	4.94	4.65	4.46	138	140	141	10.6	2
7.1	4.69	6.24	6.27	138	141	139	10.1	2
4.9	5.01	5.45	7.06	141	140	140	10.2	2
5.5	4.83	5.8	5.5	133	138	144	9.6	2
4.1	4.45	4.8	4.6	144	136	132	8.8	2
4.3	6.3	5.38	4.37	141	138	139	8.5	2
5	6.61	4.66	5.09	129	140	140	8.2	2
6.1	4.08	4.9	4	136	140	142	10.8	2
4.7	3.3	4.23	4.09	141	138	140	8.5	2
	3.55			132			8.6	1
	4.09			134				1
	5.85			136				1
								1
	5.81			136			8.6	1
	5.46			138				1
	6.95			138				1
	4.22			136			12.9	1
	9.2			131				2
	4.05			140			9.3	2
	6.56			138				1
	5.98			133				1
	5.32			135				1
	1.62			133				1
	5.5			141				1
	3.92			142			10.8	2

	5.5			141				1
	7.45			142				2
4.9	5.01	5.45	7.06	141	140	140	8.9	2
	4.08			138			8.4	2
3.4	31.7	3.65	4.12	134	140	143	8.2	2
5.9	5.21	5.74	6.12	137	143	139	7.8	1
2.7	5.25	4.5	5.28	137	138	138	10.2	2
3.3	3.3	4.89	5.11	138	138	139	5.1	1
2.5	2.51	5.23	5.4	145	137	142	8	2
4.4	4.36	4.46	5.42	138	143	141	7.7	2
	3.43			132			9.4	2
4.9	4.66	3.88	5.04	142	139	141	8.7	2
	3.96			143			10.1	1
3.9	3.86	3.95	4.43	124	141	141	32	2
4.6	5.98	5.02	7.21	139	142	141	7.9	2
3.7	7	5.21	3.9	137	140	144	6.7	2
6.3	3.87	6.84	6.75	134	139	141	9.8	2
5.8	4.87	4.67	5.3	140	136	138	9.4	2
5.3	4.91	4.95	4.84	137	141	140	10.9	2
5	4.96	4.85	5.07	144	141	143	10.2	2
4.2	5.21	5.6	5.1	137	139	139	10.2	2
3	4.82	5.22	4.46	138	141	143	6.7	2
4.8	5.4	7.53	4.7	135	141	140	8.2	2
4.9	4.59	5.94	5.97	135	141	140	9.5	2
3.7	5.04	4.31	5.04	135	141	144	9.4	2
4.8	5.04	4.62	6.17	138	143	139	8.2	2
5.4	5.9	5.35	5	142	144	141	7.6	2
	4.1	5.37		134	134		8.4	2
4.5	5.8	3.93	5.9	137	139	140	10.2	2
5.5	4.09	6.01	5.76	127	136	141	10.2	2
4.8	6.23	5.33	5.01	144	143	138	9.4	2
2.3	4.43	5.28	4.9	137	139	136	10.2	2
6.6	4.44	6.95	6.4	136	137	145	11.4	2
5.2	7.31	5.43	5.93	141	144	142	10.4	2
4.2	7.1	1.56	4.93	132	136	139	11.2	2
5.8	5.02	5.88	5.9	135	138	141	9.8	2
2.7	5.81	5.18	3.9	139	142	143	2.5	2
4.6	5.75	5.77	5.08	138	137	138	9.5	2
4.8	7.19	6.83	6.4	132	135	136	10.4	2
6.2	6.54	5.65	5.38	144	139	141	10.4	2
5.9	3.96	6.3	6.9	144	139	141	10.9	2
5.2	5.46	5.43	6.73	141	141	142	10	2
5.2	5.14	6.26	6.88	139	142	145	10.6	2
5.8	4.98	5.83	5.91	139	142	144	10.9	2
5.9	5.51	5.61	6.16	144	142	141	9.8	2
5.2	4.91	5.27	6.39	142	144	141	9.2	2
4	4.81	5.61	5.72	133	140	132	9.8	2

5.1	5.87	4.49	6.75	144	140	145	10.8	2
5.4	5.58	4.85	5.91	142	141	137	10.2	2
5.7	5.28	5.99	5.95	138	138	144	10	2
4.5	5.38	5.82	5.92	138	143	141	10.7	2
2.9	5.27	5.4	5.99	138	139	140	9.8	2
6.3	5.55	4.88	5.58	141	144	142	10.8	2
5.7	5.2	4.7	5.91	138	139	140	9.7	2
5.1	5.72	5.49	4.98	139	143	142	10.3	2
4.9	5.59	6.03	5.1	138	136	140	10.3	2
4.2	4.7	4.82	5.63	140	143	141	8.6	2
3.6	5.42	4.95	5.19	143	138	144	11.2	2
4.7	5.43	4.89	5.92	140	147	144	6.8	2
3.9	6.04	5.63	5.01	141	147	149	4.8	2
2.9	4.03	5.56	5.16	128	139	134	9.4	2
5.5	5.31	5.05	5.09	139	138	140	10.4	2
5.1	5.95	5.19	5.9	138	131	139	9.2	2
5.8	7.53	5.46	6.91	137	143	140	9.5	2
4.6	5.96	5.02	5.49	140	144	142	10.8	2
5.2	6.01	5.45	0.3	134	135	139	10.2	2
6.5	5.17	4.29	5.79	135	141	147	10.2	2
	6.38			125			8.2	2
	7.3	5.76		136	145		10.4	2
	6.9	6.12		140	145		10.1	2
5.2	6.77	5.19	5.09	136	144	147	9.5	2
5.7	5.71	5.91	5.09	128	137	140	10	2
2.5	4.99	5.61	5.71	139	140	147	10.4	2
4	3.52	8.7	3.53	148	141	158	9.5	2
5.3	6.16	6.54	4.34	124	138	126	10.2	2
6.2	4.59	4.71	5.19	140	144	142	9.8	2
4.8	4.74	5	5.36	136	142	145	10.3	2
	5.64			125			9.6	2
6.7	4.9	5.95	6.73	140	142	141	9.5	2
	5.19	4.53		141	141		9.8	2
	4.95	5.9		136	137		8.7	2
6.7	4.13	5.5	6.19	144	148	148	9.2	2
6.1	5.51	5.91	6.72	145	144	148	5.7	2
	8.84			146			9.4	2
	3.53			158			8	2
	5.9			127			10.3	2
	5			129			9.2	2
	4.85			132			9.8	2
	6.78			125				2
	7.72			148			10.2	2
	5.42			144			10.4	2
	4.65			141			5	2
	5.48			144				2
	5.04			140			9.2	2

2.7	2.41	3	4.19	129	131	137	8.9	2
4	5.3	5.9	5.52	146	135	144	6.6	2
5	4.42	5.3	5.91	134	146	140	9.6	1
5.3	5.29	4.07	4.72	124	131	136	10.7	2
5	5.7	5.1	4.07	134	134	141	9.8	2
4.8	5.2	5.65	4.35	141	137	139	10.2	2
5	4.07	5.2	5.3	142	141	147	10.9	2
3.7	5.1	4.2	5.1	146	146	131	10.2	2
3.2	5.7	4.2	5.9	140	132	147	9.8	2
	6.03			143			10	1

HIV	FDate_F75	time_F75	F75_days	Refeeding	Date_refee	Time
2	########	12:00:00	9	1	########	
2	########	04:00:00	4	2		
2	########		8	2		
2	########		16	1		
2	########		8	1	########	
2	########		16	2		
2	########		6	1	########	
2	########		6	2		
2	########		12	1		
2	########		17	2		
1	########		9	2		
2	########		16	1	########	
2	########		7	2		
2	########		6	2		
2	########		23	2		
2	########		12	2		
2	########		12	2		
2	########		12	2		
2	########		4	2		
2	########		7	1	########	
2	########		5	2		
2	########		8	2		
2	########		7	2		
2	########		6	2		
2	########		16	2	########	
2	########		23	2		
2	########		14	1	########	
2	########		20	1	########	
2	########		5	2		
2	########		13	1	########	
2	########					
	########					
2	########					
2	########					
1	########					
2	########					
2	########					
2	########					
2	########					
2	########					
2	########					
2	########					
2	########					
2	########					
2	########					
2	########					

2	########			
2	########			
2	########	6	2	
2	########			
1	########	11	2	
2	########	8	2	
2	########	16	1	########
2	########	10	2	
2	########	9	1	########
2	########	20	1	########
2	########			
2	########	9	2	
1	########			
2	########	9	2	
2	########	10	1	########
2	########	8	1	########
2	########	17	2	
2	########	9	2	
2	########	21	1	########
2	########	11	2	
2	########	16	1	########
2	########	9	2	########
2	########	7	1	########
2	########	15	2	
2	########	12	1	########
2	########	12	1	########
2	########	7	2	
1	########	8	2	
1	########	20	1	########
2	########	9	2	
2	########	11	2	
2	########	9	2	
2	########	14	2	
2	########	15	2	
2	########	9	1	########
2	########	5	2	
2	########	23	1	########
2	########	7	1	########
2	########	5	2	
2	########	5	1	########
2	########	4	2	
2	########	8	1	########
2	########	8	1	########
2	########	13	1	########
2	########	21	2	
2	########	17	1	########
2	########	9	1	########

2	########	12	2	
2	########	7	2	
2	########	3	2	
2	########	10	2	
2	########	12	1	########
2	########	6	1	########
2	########	5	2	
2	########	4	1	########
2	########	4	1	########
2	########	5	2	
2	########	5	1	########
2	########	16	2	
2	########	9	1	########
2	########	14	2	
2	########	6	2	
2	########	13	2	
2	########	7	2	
2	########	14	2	
2	########	7	2	
2	########	12	2	
2	########			
2	########	10	2	
2	########	10	1	########
2	########	6	2	
2	########	7	2	
2	########	7	1	########
2	########	12	1	########
2	########	6	2	
2	########	5	2	
2	########	9	2	
2	########			
2	########	6	2	
2	########	5	2	
2	########	5	2	
2	########	8	2	
2	########	9	2	
2	########			
2	########			
2	########			
2	########			
1	########			
2	########			
2	########			
2	########			
2	########			
2	########			
2	########			

2	########	6	2	
2	########	4	2	
2	########	7	2	
2	########	5	2	
2	########	5	2	
2	########	9	2	
2	########	7	2	
2	########	19	2	
2	########	5	1	########
2	########			
